# Patients Perceived Knowledge, Attitude, and Practice of Dental Abscess Management in Periurban District, Ghana

**DOI:** 10.1155/2022/2266347

**Published:** 2022-06-20

**Authors:** Kingsley Adeoye Damilare, David Abass, David Antwi-Agyei, Frederick Osei-Owusu, Ebenezer Ahenkan, Kwame Adu Okyere Boadu, Richard Okyere Boadu

**Affiliations:** ^1^Effiduase Government Hospital, Ghana Health Service, Ghana; ^2^Kumasi Centre for Collaborative Research in Tropical Medicine, Kwame Nkrumah University of Science and Technology, Kumasi, Ghana; ^3^School of Medicine and Dentistry, College of Health Sciences, Kwame Nkrumah University of Science and Technology, Kumasi, Ghana; ^4^Department of Health Information Management, School of Allied Health Sciences, College of Health and Allied Health Sciences, University of Cape Coast, Cape Coast, Ghana

## Abstract

**Background:**

Seeking conventional oral health care for various dental conditions is still a challenge for many worldwide and poses a global public health challenge. It is not until the individual starts experiencing pain and disfigurement of the facial profile that they seek dental care after many self-care interventions have failed. This study sought to determine perceived knowledge, attitude, practice, and satisfaction of dental abscess management among dental abscess patients (DAPs).

**Methods:**

The research used a cross-sectional design to collect data from patients who visited the Dental Department of Effiduase Government Hospital with dental abscess from 6 May 2020–27 August 2020. A total sample size of 82 was selected from a population of 377. About 76 DAPs who met the inclusion criteria and consented to participate were interviewed.

**Results:**

About 57% of respondents believed that dental caries caused their abscess. However, 46% sought medical treatment between 0 and 2 weeks after the onset of their dental abscess, while the rest did that after 3 weeks. Close to 64.5% have ever had episode of abscess before current one which they managed with alternative treatment. About 95% of respondents saw the hospital as a good place for dental care. A significant association was established between patient's level of education and influence to seek orthodox treatment (*p* = 0.015, Fisher's exact test (FET)). Another significant association was established between patient's level of education and the kind of alternative treatment they used (*p* = 0.016, Fisher's exact test (FET)).

**Conclusions:**

Most DAPs believe that dental caries was the main cause of dental abscess. DAPs sought late medical care, after they have tried a failed alternative treatment. DAPs expressed satisfaction with reception, equipment and materials, and medicines used. Majority of the patients rated the cost of dental care in the facility as affordable.

## 1. Introduction

Seeking conventional oral health care for various dental conditions is still a challenge for many worldwide and poses a global public health challenge [1, 2]. It is estimated in the Global Burden of Diseases [3] that oral diseases in 2017 alone affected 3.5 billion people which was equivalent to half of the planet's human population with tooth decay in permanent teeth being the commonest condition assessed. Dental abscesses have been established to be synonymous with an accumulation of pus at the root apex of a tooth [4]. According to Shweta and Prakash [5], dental abscess is a broad term for abscesses of dental (tooth) origin. These abscesses are a periapical abscess (pus formed around the root of the tooth) and dentoalveolar abscess (when it involves both the tooth and the surrounding bone called the alveolar bone) [6]. It occurs after an infection of the pulp of the tooth by polymicrobial organisms or seepage of microorganisms, predominantly the streps species of bacteria (normal flora of the oral environment) into an exposed pulp [6, 7]. It usually occurs as a complication of dental caries, deep fillings, or failed root canal treatment and trauma [6]. After the pulp chamber has been compromised, the root canals are invaded with myriads of bacteriological agents. Furthermore, acute inflammation of the pulp commences leading to pain [7]. A lot of self-care interventions by the individuals with such tooth pain are used to alleviate the pain, which in some cases kills the nerves providing some temporary relief although the infections persist [8]. With time, when the immunity of the individual is compromised, the infection progresses to a dental abscess which may be either periapical or dentoalveolar abscesses or in some life-threatening cases, the infection progresses to the potential facial spaces of the jaws with associated complications such as cellulitis, cavernous sinus thrombosis, osteomyelitis, and Ludwig angina [6]. It is not until the individual starts experiencing pain and disfigurement of the facial profile (facial asymmetry) that they seek dental care after many self-care interventions have failed [9].

According to Opeodu et al. [10], health-seeking behaviour is a key indicator for assessing issues related to health promotion globally. Though many factors have been enumerated to be contributing to patient's behaviour in seeking early dental care, notable among them as cited by the World Health Organization are accessibility, transportation, awareness, financial, and psychosocial factors [11]. In addition, inadequate health resources, the attitude of healthcare personnel, and lack of health information among others are also considerable factors that could influence patients' health-seeking attitudes to oral health care [12, 13]. Delays in dental care have also been attributed to dental fear, dental pain, cultural beliefs, lack of dental care facilities, and unavailability of dental care professionals among other healthcare factors [14].

Complex associations exist among beliefs about oral health care, socioeconomic status (SES) in early life, and oral health-related behaviours [15]. Reports on the state of oral health of the United States population suggest that people from specific ethnic minorities often have poor oral health status. Being a part of an ethnic minority group does not inevitably lead a person to have poor oral health. It does suggest, however, that there may be certain cultural beliefs and practices common to the people in these groups which influence their oral health status, such as values placed on having healthy primary teeth or expectations about preventive or therapeutic interventions [16]. Beliefs and cultural factors may have important implications for an individual's own health and those of others for whom they provide care, such as children and the elderly. Culture is often defined as coherent, shared patterns of actions or beliefs specific to named groups of people that provide basic life roadmaps or social contexts, defining behavioural norms and interpersonal relationships as well as unwritten rules for proper living.

The generational beliefs in oral health also stem from the importance of the mouths to both their overall health and social lives [16]. The frequency of untreated carious and missing tooth surfaces was linked to oral health-related quality of life [17]. Since childhood, oral health has been linked to intergenerational factors and many aspects of people's beliefs, dentist attendance, and self-care [18]. There are several studies on oral health beliefs, myths, cultural beliefs, values, and practices among different cultural groups. Studies conducted among the population of the United States found that ethnic minorities have poor oral health status [19]. The reason for poor health is because the cultural beliefs and practices commonly followed among these groups influence their oral health status. According to Nguyen and colleagues [20], in Vietnam communities, beliefs in traditional oral dental care are preferred over Modern orthodox treatment. The study also supported existing literature that Vietnamese oral health beliefs and practices impact the use of Western health care services.

In a cross-sectional study conducted by Ren et al. [21], the level of oral health knowledge, beliefs, and practices among older adults in Shanghai, China, was not encouraging. However, younger and working individuals with a higher level of education showed good oral health knowledge, beliefs, and practices. Low patronage of orthodox dental care in some communities in sub-Saharan Africa has been reported, as confirmed by Uguru N et al., [22] in Nigeria. This shows that the level of awareness and acceptability of conventional dental care in many low- and middle-income countries is low. Considering the low patronage of standard dental care, this study sought to understand the dynamics involved in patients with dental abscess health-seeking behaviour toward dental care and also to decipher why individuals stay for a long time, after experiencing toothache, before seeking dental care considering the complications that come with toothache. We sought to determine perceived knowledge, attitude, practice, and satisfaction among DAPs. We further sought to test hypotheses of probable association between background characteristics of patients and their knowledge, attitude, and practice of dental abscess management.

## 2. Methods

### 2.1. Study Design

The research used a cross-sectional design to collect data from patients who visited the dental department with dental abscess from 6 May, 2020–27 August, 2020. This design sought to determine health-seeking behaviour of the targeted patients.

### 2.2. Profile of Study Areas

Effiduase Government Hospital is a Ghana Health Service (GHS) primary healthcare facility with 53 bed capacity under the Sekyere East district of the Ashanti region of Ghana. The hospital serves residents of the district with a total population of 77,365 and also receives referrals from four subdistricts and parts of neighbouring sister districts such as Sekyere Kumawu and Sekyere South. It is in the heart of the town, a few kilometres from the town's busy market. Built and operated as health center in 1954 and later converted to district hospital status in 1996, it provides basically preventive and curative medicine.

### 2.3. Study Population

The study population was 377 representing expected dental outpatient attendants during the period of study in Effiduase Government Hospital.

### 2.4. Sample Size Determination

A total sample size of 82 (cluster size = 1) was selected from a population of 377 using StatCalc function in Epi Info 7 software (confidence level = 80%, expected frequency = 20%, acceptable margin of error = 5%, design effect = 1, and cluster = 1).

### 2.5. Sampling Procedure

Effiduase Government Hospital was purposely selected, due to concerns regarding the patients' delay in seeking early dental care in the facility before it complicates to a dental abscess. Patients who visited the dental OPD with dental abscess were selected. This process continued until the required respondents were sampled. Those who were in critical conditions and unconscious were excluded from the study. About 76 dental outpatients who met the inclusion criteria and consented to participate were interviewed.

### 2.6. Data Collection and Analysis

We developed a structured questionnaire that reflected the specific objectives of the study. The questionnaire which comprised three parts was used to solicit information from respondents. The first part included questions regarding sociodemographic data such as gender, age, marital status, education, and occupation. The second part assesses the patient's knowledge, attitude, and practice of dental abscess management. In the third part, the patients' satisfaction of patients' perceived quality of care in previous visited dental care facility were evaluated using a 7-point scale. Each action statement or item related to these dimensions was assessed using a Likert scale of agreement, ranging from one (very dissatisfied) to seven (very satisfied). This enabled the participants to decide about the intensity of their satisfaction or dissatisfaction. To simplify, the analysis scales denoting satisfaction were added together as “satisfied,” whereas the scales representing dissatisfaction were added as “dissatisfied.” A collection of items belonging to patients' perceived quality and their ratings was added together and divided by the total number items and multiplied by one hundred to create an overall percentile score. The questionnaire was administered with the assistance of registered dental surgery assistants and volunteer nurses from the hospital. The data collectors guided the respondents who could not read or understand the English language to answer the questionnaire to ensure the quality of data collected.

### 2.7. Reliability and Validity

The questionnaire for the study was pretested at Nkwakwanua Health centre, which is not part of the study facility but has common background characteristics. Based on feedback from the field pretest, the tool was modified to ensure its suitability for the study. Research assistants were given 3-day training on the research protocol and data collection tools. This was important to make them familiar with the tools and the expected way of questionnaire administration to reduce inconsistencies and biases. To ensure data quality, data verification was conducted for randomly selected administered questionnaires. Also, data validation checks were included in the data entry software to minimize data entry errors.

### 2.8. Hypothesis

We tested the null hypothesis that there is no association between the background characteristics of patients and their knowledge, attitude, and practice of dental abscess management. The independent variable used for the test included background characteristics of the patient such as gender, age, marital status, education, religion, and occupation, and the dependent variables comprised of knowledge, attitude, and practice of dental abscess management are independent. To determine if there was a statistically significant association between the independent and the dependent variables, Fisher's exact test was performed in SPSS. The null hypothesis for Fisher's exact test is that the two variables are independent, which is a two-sided test; so if the *p* value is not less than 0.05, we do not reject the null hypothesis.

### 2.9. Ethical Considerations

We sought institutional approval for the study. An introductory letter from the Department of Health Information Management, University of Cape Coast, was sent to the facility. The letter explained the purpose of the study as well as the reason for collecting the data. Approval was given by the hospital management. Respondents were assured of their confidentiality of the information they would be providing. The purpose of the study and the various sections of the questionnaires were explained to respondents to enable them to answer the questions conveniently.

## 3. Results

### 3.1. Sociodemographic Background of Respondents

The gender distribution of the participants comprises of majority being (61.8%) females and the rest (38.2%) males. With respect to the age distribution of participants, one person did not say his age and most (50.0%) of the respondents were young with the middle-aged patients following closely with 36.8%. Only 11.8% of respondents were elderly. In summary, the mean age was 44.6, the range was 15-98, and the standard deviation was 17.3 with a standard error of mean of 2.0.

With regard to marital status, while more than half of respondents (55.3%) were married, the remaining 43.4% were mostly single, widowed, divorced, or cohabiting respectfully. However, one respondent gave no response. Regarding educational status, close to 41% of respondents were in junior high school/middle school. Almost 24% of respondents had gone to the senior high/vocational/technical school. About 16% and 12% had been through primary and postsecondary education, respectively, and the remaining 8% had no formal education. Substantial proportion of respondents (86%) were Christians. About 11% were Muslims, 3% were traditionalists, and 1% gave no response. The results further indicate that about 32% of respondents were farmers and almost 24% were traders. Salaried workers and unemployed folks were 13% each. Close to 4% were artisans, while almost 12% were into other occupations.

### 3.2. Treatment Support for Dental Abscess Patients

Table 1 indicates whether respondents had treatment support. While about 3% gave no response, close to 32% had no treatment support, while the 66% majority had one.

With respondents who have treatment support, half of them have one supporter, 32% have two supporters, 10% have three supporters, 4% have either five or more than five supporters, and 2% either did not respond or had four supporters (Table 1).

### 3.3. Patients' Knowledge, Attitude, and Practice of Dental Abscess Management

About 57% of respondents believed that dental caries caused their abscess, almost 15% could not attribute it to any factor, 13% thought tooth fractures caused it, 9% did not give a response, and both 5% and 1% believed it was an external trauma and bone or fish prick, respectively, as captured in Table 2(a). About 3% of respondents did not respond. In almost 4% of respondents, said the duration of their abscess lasted 9-11 weeks, whereas 7% said 6-8 weeks. For 3-5 weeks and 12 or more weeks, the respondents involved were 13% and 28%, respectively, but 46% had it for less than 3 weeks (Table 2(a)). Interestingly, about 65% had had a previous episode of abscess, while 33% had never had an episode in their lives. Of the respondents, 3% gave no answer to this (Table 2(a)).

Of respondents with previous episodes, 43% self-medicated on orthodox drugs, while 22% resorted to herbal medication. Even though 16% resorted to other treatment options, 12% did not have treatment on mind, 4% had no response, and the remaining 2% did homeopathy (Table 2(a)). The treatment methods proved futile for 29% of respondents, but 67% had positive outcome after usage. About 2% had no idea on the effect of their treatment mode did (Table 2(b)). Of the respondents as stated in Table 2(b), 63% were quick to seek alternative treatment within 3 months, while a little above 22% did that after 12 months. About 6% and 2% of respondents sought treatment within 10-12 months and 4-6 months, respectively. However, about 6% could remember when they sought treatment.

An overwhelming number of 82% were influenced to seek orthodox dental care. Just 18% of respondents were not influenced in (Table 2(b)). Interestingly, about 45% of respondents in (Table 2(a)) were convinced by family members to seek treatment, while 31% were self-motivated to seek treatment. Almost 12% were convinced by their peers, 7% by health workers, and close to 5% by other people. While 1% and about 4% have been seeking orthodox medication for 10-12 months and 4-6 months, respectively, about 20% have been doing it for more than 1 year, while 22% did not know the exact duration in (Table 2(b)). More than half of respondents (about 53%) had been seeking orthodox medication for less than 3 months.

The results suggest that there is no association between background characteristics (such as gender, age, marital status, religion, and occupation) of patients and their knowledge, attitude, and practice of dental abscess management. However, a significant association was established between patient's level of education and influence to seek orthodox treatment (*p* = 0.015, Fisher's exact test (FET)). Furthermore, another significant association was established between patient's level of education and the kind of alternative treatment they use (*p* = 0.016, Fisher's exact test (FET)) (not indicated in the table).

### 3.4. Perceived Quality of Care in Previous Visited Dental Care Facility

Almost all (95%) of respondents saw the hospital as a good place for dental care. A little above 1% each did not see it that way or did not know at all. Close to 3% did not respond to this question. During dental care, 78% of patients were very satisfied and 20% were just satisfied. The remaining people who were a little above 2% did not give any response. With 1% of the population being very disappointed with the equipment and materials used for their dental care, about 24% were satisfied, 67% were very satisfied, and those who did not respond were about 8%. Of the prescribed medications, about 59% were very satisfied, a little above 30% were satisfied, 9% gave no response and closely above 1% were somewhat satisfied.

Health insurance caters for the dental care of a little above 38% of respondents and 30% fund their own dental care. While about 22% are funded by relatives, close to 3% having other means of funding were not stated and close to 7% gave no response. Interestingly, about 47%, 34%, and 4% rated the cost of dental care in the facility as very affordable, affordable, and somewhat affordable, respectively. About 8% gave no response, about 3% thought it was expensive, and a little above 1% each felt the rate was neither affordable nor expensive, somewhat expensive, or very expensive. With regard to competency of staff, about 5% did not comment on it, while about 26% thought the staff were competent and a little above 68% highly rated the competency of the staff. About 86% of respondents were very likely to recommending the hospital to people with dental abscess, while 9% were likely, and the remaining gave no response.

## 4. Discussions

This study seeks to gain an understanding of the dynamics involved in dental abscess patients' health-seeking behaviour toward dental care and also why individuals stay home for a long time, after the onset of the ailment, before seeking dental care considering the hitches that come with a toothache. The study sought to determine perceived knowledge, attitude, practice, and satisfaction among DAPs. Also, the study tested the hypotheses of a possible association between background characteristics of patients and their knowledge, attitude, and practice of dental abscess management. There is scanty literature on dental abscess in sub-Saharan Africa, particularly in Ghana. The findings of this study to a large extent will contribute to the generation of further knowledge, the creation of awareness of the problem among different health stakeholders which is expected to help in establishing more realistic interventions for dental health education, and the promotion of dental health through oral health outreaches and community sensitization.

### 4.1. Sociodemographic Background of Respondents

Oral disease conditions have a higher incidence in females compared to males [22]. Bruce et al. [23] attributed the higher prevalence of dental diseases in females to a higher sugar intake. In addition to this is females having a higher attendance for healthcare interventions including the use of hospital-based treatment. Our findings buttress this assertion to the fact that over 6 out of every 10 patients who reported to the dental clinic with dental abscesses were females. However, Lipsky et al. [24] queried the lower prevalence of dental disorders in males by attributing it to habitual avoidance of hospitals. Age is an important determinant in dental health. Paediatrics, teenagers, and young adults have been confirmed to have higher incidence and prevalence of dental caries which could further complicate to dental abscesses, while older adults are prone to periodontal diseases [25]. Again, our findings support this claim, for instance half of respondents who visited the dental clinic with dental abscess cases were young adults. Studies show that education and occupation correlate with either higher or lower intake of sugar and also correspond to seeking oral care. According to Vano et al. [26], people on the higher ladder of education and higher income earners take more refined foods but are conscious of their oral health. In addition, their health-seeking behaviour encompasses regular dental check-ups than people with less education and low-income occupation, thus limiting their vulnerability to getting affected by dental abscess. Oral health knowledge is linked to the educational degree of the individual subject as reported by Márquez-Arrico et al. [27]. However, oral health knowledge does not always translate into healthy habits.

### 4.2. Treatment Support for Dental Abscess Patients

Caregiver support is highly essential in management of patients with dental abscess. Caregivers are reported to play critical roles in nurturing, devotion of time, and financial support to as meaningful attributes towards care of patients [28]. In the event of patients being admitted for further management of dental abscess, caregiver's support would critically play a role alleviating the pains patients with dental abscess go through. Coincidentally, more than half of the patients interviewed had at least one person who offer them treatment support. Nevertheless, there is the need to educate the general public especially families of patients on the importance of caregiving in the management of dental abscess.

### 4.3. Patients' Knowledge, Attitude, and Practice in Dental Abscess Management

According to [6], a report by National Center for Health Statistics for dental caries and tooth loss found that 91% of persons aged 20 to 64 had dental caries that progressed to a dental abscess which buttresses the fact that a significant proportion of dental abscess is caused by dental caries. This is consistent with several studies which have established dental caries as the main source of infection progressing to dental abscess. Our study reveals that almost six out of every ten dental abscess patients believed that dental caries caused their abscess, which is consistent to what Sanders and Houck established. However, the remaining attributed the cause to other factors such as tooth fractures, external trauma, and bone or fish prick. A dental abscess takes an average of one to two weeks to heal [6]. Dental abscesses could be chronic, thus reoccurring in a number of clients depending on the types of microorganism involved and the status of individual's immunity [29]. A little below half of the patients interviewed had their dental abscess between 0-2 weeks prior to seeking medical care while the reaming reported after 2 weeks of episode. This is a worrisome situation given the average duration of time dental abscess lasted before seeking medical therapeutics. This suggests that majority of patients report late when they have reached chronic stage. In a study by Bayetto et al. [30], patients with dental abscesses frequently present to their primary healthcare doctors first, especially if they have a fear of dentists or are financially inadequate. Hence, clients delay their search for conventional interventions after several self-care measures have failed and have started experiencing discomfort and disfigurement of the face [9].

Dentists should have a good understanding of the various types of traditional methods of medication that people employ. It is critical for dental practitioners to understand the types of prehospital therapy that their patients take before visiting the hospital. Guo et al. [31] have suggested that by knowing and acknowledging the use of traditional medicine, dental practitioners can create a supportive atmosphere for patients to disclose their use of other alternative treatments, such as traditional medicine, and educate them appropriately. Our study revealed that six out of every ten patients resorted to self-medicated, while two out of every ten of them resorted to herbal medication prior to the visit. Though this is an interesting event for further interrogations, this study missed the opportunity to capture the types of alternative medicine used by patients. Nevertheless, some studies have suggested that some herbs and herbal preparations are helpful in the treatment of some oral conditions [32, 33]. However, those studies could not establish the efficacy of herbal medication or any other alternative treatment used for treatment of any oral diseases. There appears to be no association between general background characteristics of patients and their knowledge, attitude, and practice of dental abscess management. However, a significant association was established between patient's level of education and influence to seek orthodox treatment. Additionally, another significant association was established between patient's level of education and the kind of alternative treatment they use. Our findings support the assertion that individuals from advanced communities who are well educated compared to people of low socioeconomic status and education had perceived oral health beliefs, which had a significant association with their traditional practices and their oral health. Therefore, good oral behaviour is associated with intergenerational beliefs and formal oral health education [18].

### 4.4. Perceived Quality of Care in Previous Visited Dental Care Facility

The first five most important elements of dental professionalism, according to Taibah [34], are adherence to sterilization and infection control rules and procedures, personal hygiene and clean professional attire, good communication skills, diagnostic and clinical judgment, and provision of the most efficient dental treatment and ethical considerations. Conforming to the aforementioned qualities, almost all of dental abscess patients saw the hospital as a good place for dental care. Almost all of the patients who visited the dental hospital were satisfied with the reception accorded to them by hospital staff. Besides, nine out of every ten patients were satisfied with the equipment and materials used for their dental care. Furthermore, about nine out of every ten patients were satisfied with medications prescribed for their dental abscess. The use of dental services has been pointed to have substantial financial impact on clients and their families [35]. Health insurance for dental health care may reduce economic inequality and could assist in offsetting these effects [36]. A little over five out of every ten patients paid out of pocket whiles almost 4 out of every ten of them use national health insurance to access dental care. Over eight out of every ten patients rated the cost of dental care in the facility as affordable.

### 4.5. Limitations of the Study

The study purposely selected a hospital in Ghana; thus, it cannot be used for generalization. The types of treatment intervention used by the participants are not fully detailed in the study.

## 5. Conclusions

Most patients believe that dental caries was the main source of infection which progressed to their dental abscesses. The majority of dental abscess patients seek late medical care when they had reached a chronic stage, after seeking alternative treatment. There seems to be a significant association between a patient's level of education and influence to seek orthodox treatment. Additionally, there appears to be a significant association between a patient's level of education and the kind of alternative treatment they used. Almost all of the dental abscess patients saw the hospital as a good place for dental care, and they expressed satisfaction with the quality of care they received.

## 6. Recommendations

Since there was a significant association between patient's level of education and influence to seek conventional treatment, it is highly recommended that oral health policy should be formulated to include outreaches to catchment communities of Effiduase Government Hospital. This would enhance awareness of modernized preventive measures among communities with low level of education. Communities with very low oral health professionals should be well resourced with adequate human resources and incentives so as to enable wider coverage of outreach and oral health campaigns among deprived communities.

## Figures and Tables

**Table 1:** Treatment support.

Indicator/response	Frequency	Percent
*Do you have any treatment supporter?*		
Yes	50	65.8
No	24	31.6
No response	2	2.6
*Total*	*76*	*100.0*
*If yes, what is the number of carers/treatment supporters?*		
One	25	50.0
Two	16	32.0
Three	5	10.0
Four	1	2.0
Five and above	2	4.0
No response	1	2.0
*Total*	*50*	*100.0*

Source: 2020 survey.

**(a):** 

Indicator/response	Frequency	Percent
*In your opinion, what was the cause of your dental abscess?*		
External trauma	4	5.3
Bone or fish prick	1	1.3
Dental caries	43	56.6
Tooth fracture	10	13.2
Other	11	14.5
No response	7	9.2
*Total*	*76*	*100.0*
*Can you tell us the duration of your abscess?*		
0-2 weeks	35	46.1
3-5 weeks	10	13.2
6-8 weeks	5	6.6
9-11 weeks	3	3.9
12+ weeks	21	27.6
No response	2	2.6
*Total*	*76*	*100.0*
*Have you ever had episode of abscess before this current one?*		
Yes	49	64.5
No	25	32.9
No response	2	2.6
*Total*	*76*	*100.0*
*If yes, what kind of treatment did you use?*		
No treatment	6	12.2
Herbal	11	22.4
Self-medication	21	42.9
Homeopathy	1	2.0
Other	8	16.3
No response	2	4.1
*Total*	*49*	*100.0*

**(b):** 

Indicator/response	Frequency	Percent
*Did that mode of treatment resolve the dental abscess?*		
Yes	33	67.3
No	14	28.6
Do not know	2	4.1
*Total*	*49*	*100.0*
*How long did it take you to seek alternate treatment?*		
0-3	31	63.3
4-6	1	2.0
10-12	3	6.1
Above 12	11	22.4
Do not know	3	6.1
*Total*	*49*	*100.0*
*Were you influenced to seek for orthodox dental care?*		
Yes	42	81.6
No	9	18.4
*Total*	*49*	*100.0*
*If yes, who convinced you to seek treatment?*		
Self	13	31.0
Family	19	45.2
Peers/friend	5	11.9
Health workers	3	7.1
Other	2	4.8
*Total*	*42*	*100.0*
*How long have you been seeking orthodox medical treatment?*		
0-3	40	52.6
4-6	3	3.9
10-12	1	1.3
Above 12	15	19.7
Do not know	17	22.4
*Total*	*76*	*100.0*

Source: 2020 survey.

## Data Availability

Authors can make data available on request through a data access of the hospital administration.

## Supplementary Materials

Supplementary Materials Dental Abscess data collection tool was used to assess health-seeking behaviour of clients with dental abscess.
