# Supplementary material for: Patients Perceived Knowledge, Attitude, and Practice of Dental Abscess Management in Periurban District, Ghana
Source: Biomed Res Int. 2022 Jun 20;2022:2266347. doi: 10.1155/2022/2266347 (PMC9236784; doi:10.1155/2022/2266347)
Supplement: Supplementary Materials — Dental Abscess data collection tool was used to assess health-seeking behaviour of clients with dental abscess. [file 2266347.f1.pdf]

GHANA HEALTH SERVICE
EFFIDUASE GOVERNMENT HOSPITAL
DENTAL DEPARTMENT

Health Seeking Behaviour of Clients with Dental Abscess in Effiduase Government Hospital

QUESTIONNAIRE FOR CLIENTS

Introduction

Good morning/afternoon. I am with the Dental Department of Effiduase Government Hospital. I will be conducting interviews with clients like you to find out your views and ideas about "**Health seeking behaviour of clients with dental abscess**". Your opinion is highly essential at the same time vital as it will help us to improve the kind of service we provide to our patients. Whatever you say will be treated confidential, so feel at ease to express your candid opinion**.** Be assured that your responses will not in any way be linked to your identity. You are kindly requested to answer the questions below by indicating a tick or writing the appropriate answer when needed. **You are a volunteer. You can choose not to take part and if you join, you may quit at any time. There will be no penalty if you decide to quit the study**.

THANK YOU

BACKGROUND INFORMATION

1. Gender
   a. Male   b. Female

2. Age ....

3. Marital status
   a. Single b. Cohabitation c. Married d. Divorced or separated e. Widowed

4. Which community do you resides? …………………………………..

5. Education
   a. None b. Primary c. JSS/Middle School d. SHS/Voc/Tech   e. Post Sec/ Tertiary

6. Religion
   a. Traditional b. Christian c. Muslim Others (specify)________________

7. Ethnicity _____________

8. Occupation
   a. Housewife b. Farmer c. Trader d. Artisan e. Salaried worker f. Unemployed g. Others(specify) _______

9. Do you have any treatment supporter? a. Yes b. No

10. If yes, what is the number of carers/treatment supporters
a. One b. Two c. Three d. Four e. Five and above

KNOWLEDGE ATTITUDE AND PRACTICE

11. In your opinion, what was the cause of your dental abscess?
a. External trauma b. Bone or fish prick c. Dental caries d. Tooth fracture e. Spiritual f. Others (specify)_______________

12. Can you tell us the duration of your abscess?
a. 0-2 weeks b. 3-5 weeks c. 6-8 weeks d. 9 - 11 weeks e. above 11 weeks

13. Have you ever had episode of abscess before this current one? a. Yes b. No

14. If yes, what kind of treatment did you use?
a. No treatment b. Herbal c. Self-medication d. Spiritual/prayer e. Homeopathy f. Others (specify) ___________________

15. Did that mode of treatment resolve the dental abscess? a. Yes b. No c. Don't know

16. How long did it take you to seek alternate treatment?
a. 0- 3 month b. 4-6 months c. 7-9 months d. 10-12 months e. Above 12 months

17. Were you influenced to seek for orthodox dental care? a. Yes b. No

18. If yes, who convinced you to seek treatment?
a. Self b. Family c. Peers/Friend d. Health workers e. Others (specify) ___________________

19. How long have you been seeking orthodox medical treatment?
a. 0- 3 month b. 4-6 months c. 7-9 months d. 10-12 months e. Above 12 months

PERCEIVED QUALITY OF CARE IN PREVIOUS VISITED DENTAL CARE FACILITY

20. Do you see hospital as a good place for dental care? a. Yes b. No c. Don't know

21. Are you satisfied with the reception of the staff during dental care visits? a. Very satisfied [1] b. satisfied [2] c. somewhat satisfied [3] d. neither satisfied or dissatisfied [4] e. somewhat dissatisfied [5] f. dissatisfied [6] g. very dissatisfied [7]

22. Are you satisfied with the equipment and materials used during your dental care visits? a. Very satisfied [1] b. satisfied [2] c. somewhat satisfied [3] d. neither satisfied or dissatisfied [4] e. somewhat dissatisfied [5] f. dissatisfied [6] g. very dissatisfied [7]

23. Do you think medications prescribed for your dental abscess helped/is helping?
a. Very satisfied [1] b. satisfied [2] c. somewhat satisfied [3] d. neither satisfied or dissatisfied [4] e. somewhat dissatisfied [5] f. dissatisfied [6] g. very dissatisfied [7]

24. How are you currently financing your dental care?

a. Self b. Relative c. Health insurance d. Neighbour/ Peer/friend e. Others (specify) ___________________

25. In your opinion, how do you rate the cost of dental care in the facility?

a. Very affordable [1] b. affordable [2] c. somewhat affordable [3] d. neither affordable or expensive [4] e. somewhat expensive [5] f. expensive [6] g. very expensive [7]

26. How will you rate the competency level of staff on dental care?

a. Very competent [1] b. competent [2] c. somewhat competent [3] d. neither competent or incompetent [4] e. somewhat incompetent [5] f. incompetent [6] g. very incompetent [7]

27. How likely will you recommend the hospital for someone with dental abscess?

a. Very likely [1] b. likely [2] c. somewhat likely [3] d. neither likely or unlikely [4] e. somewhat unlikely [5] f. unlikely [6] g. very unlikely [7]
